# Structural Color Controllable Humidity Response Chiral Nematic Cellulose Nanocrystalline Film

**DOI:** 10.3390/bios12090707

**Published:** 2022-09-01

**Authors:** Ran Duan, Mengli Lu, Ruiqi Tang, Yuanyuan Guo, Dongyu Zhao

**Affiliations:** School of Chemistry, Beihang University, Beijing 100190, China

**Keywords:** cellulose nanocrystal, poly(ethylene glycol), glycerol, chiral nematic, humidity response

## Abstract

Through self-assembly, environmentally friendly cellulose nanocrystals (CNCs) can form films with a photonic crystal structure whose pitch size can be adjusted in a variety of ways at the fabrication stage. Moreover, the films exhibit response performance to multiple stimuli, which offers extensive applications. Poly(ethylene glycol) (PEG) and CNCs combine to form a smaller chiral nematic domain that develops a solid film with a uniform spiral structure when slowly dried. By changing the composition of CNCs and PEG, flexible and flat photonic composite films with uniform structural colors from blue to red are prepared. Benefiting from the change in pitch size by insertion and detachment of water molecules into the chiral nematic structure, CNCs films and CNC-PEG composite films exhibit a reversible structural color change in response to different humidity. In addition, the chiral nematic films formed by the combination of glycerol and CNCs have a reversible stimulation response to hydrochloric acid gas. Similarly, adjusting the ratio of glycerol can control the pitch size of the films and, thus, the reflective color. In summary, the pitch size of the photonic crystal structure of the films can be precisely tuned by regulating the additive ratio, and the two prepared films have reversible responses to humidity and hydrochloric acid gas, respectively. The CNC-based films show promise in the application of colorimetric biosensors.

## 1. Introduction

Many creatures in nature have unique and bright structural colors, such as butterfly wings, crustacean shells, bird feathers, and cephalopod skins [1,2]. Animals utilize them to reflect sunlight and form a variety of colorful appearances, which play important physiological roles in covert, courtship and aggressive behaviors. Different from colorant-based pigmentation, structural colors arise from the light interference in periodically layered or lattice structures, which allows them to exhibit excellent reversible responses to external stimuli [3,4,5].

Cellulose nanocrystal-based colorimetric biosensors with structural colors are very promising due to their biocompatibility, biodegradability and non-toxicity. Thus, they play an important role in environmental monitoring, medical diagnostic tools and foodstuff processing safety applications. Cellulose nanocrystal (CNC) is commonly prepared by sulfuric acid hydrolysis of natural cellulose and generally has an anisotropic rod shape (diameter 3–20 nm) with a negatively charged surface [6] so that it can be well-dispersed in water [7]. Upon evaporation, colloidal CNC suspensions spontaneously assemble into a solid film with the structure of a cholesteric liquid crystal and a structural color [8,9,10]. At the same time, the CNC film retains its left-handed band structure during evaporation, which can reflect left-handed circularly polarized light. Photonic crystals (PhCs) are spatially ordered structures with lattice parameters comparable to the wavelength of the propagating light. Their geometric and refractive index characteristics lead to an energy band structure of photons, which may allow or prohibit the propagation of electromagnetic waves in a limited frequency range. Since the periodicity of the helical structure of the chiral nematic phase of CNC matches the wavelength of visible light, CNC film can also be viewed as a kind of photonic crystal. Self-assembled photonic crystal structures made of the cholesteric liquid crystal inserted by monodisperse colloidal inorganic or polymer microspheres successfully produce new materials with structural colors that respond to external physical or chemical stimulation [11,12].

Furthermore, the introduction of additives in the colloidal CNC suspensions can obtain the tunable pitch of CNC and thus control the photonic band gap, and the additives bind to CNC nanorods, participating in self-assembly and forming chiral nematic structures. Various additives such as Si(OEt)_4_, Si(OMe)_4_, 1,2-bis(trimethoxysilyl)ethane, 1,2-bis(triethoxysilyl)ethane [13,14], monomers such as acrylamide, N-isopropylacrylamide, acrylic acid, 2-hydroxyethyl methacrylate [15,16,17], water-soluble polymers such as poly(vinyl alcohol) and poly(ethylene glycol) [18,19], silver nanowires and fluorescent latex nanoparticles [20,21] were introduced into the chiral structure of CNC. The reflection wavelength of CNC film prepared by the method of introducing additives can be tuned in the range of the visible spectrum, which provides the possibility of preparing sensitive sensors for visual recognition. Compared with visual sensors based on highly ordered nanopore inverse opal films [22,23], CNC self-assembled nanostructures have good compatibility with a variety of materials, making it easy to adjust the surface of the sensor performance, so CNC nanocomposites are expected to become a new type of structural color-based sensor application platform. As a neutral polymer with excellent compatibility and affinity for CNCs, the introduction of PEG can not only tune the structural color of CNC films but also enhance the sensitivity of the structural color of CNC films to moisture. Compared with optical fiber humidity sensors based on the change in the optical refractive index of humidity-sensitive materials, CNC-PEG film has the advantages of being inexpensive and having a simple structure as a humidity sensor.

However, a few studies show that, in addition to humidity, CNC-based nanocomposites have a broad and rapid effect on environmental stimulation [24,25]. This can be attributed to the dynamic response of the pitch size of the CNC composite film to external stimuli. Compared to conventional dyes or pigments, the structural color produced by the physical properties of light never fades. In addition, cellulose is an abundant and sustainable resource in nature and is environmentally friendly. Therefore, exploring the widespread application of CNC nanostructures as high-sensitivity visual sensors is a challenging but very attractive task.

In this work, through scanning electron microscopy (SEM), polarization optical microscopy (POM), UV–visible spectroscopy, and other comprehensive characterization methods, the CNC-PEG and CNC-glycerol composite films with response properties to humidity and hydrochloric acid vapor, respectively, are explored. Herein, through the cell ultrasonic method and the addition of PEG, the pitch of the chiral nematic phase structure in the solid film can be adjusted, and the color of the CNCs film can be controlled to obtain a composite film with a tunable photonic band gap [26]. The color of the composite film varied with the relative humidity in the range of 50%–100%. Moreover, glycerol can also be used as an additive to develop color-tunable CNC composite film, which can not only control the pitch of the film through different ratios but also grants the film the ability to respond to hydrochloric acid gas. The color of the film changes in the presence of hydrochloric acid gas, and the change is reversible. In summary, we demonstrate the facile and scalable colorimetric biosensors, indicating broad application prospects of CNC-based film as versatile, low-cost, reusable and high-sensitivity analytical methods used to monitor potential hazards or changes in the surrounding environment.

## 2. Materials and Methods

### 2.1. Materials

Microcrystalline cellulose (MCC), PEG and dialysis bags, molecular weight 800–14,000, were purchased from Sinopharm Chemical Reagent (sinoreagent.com, accessed on 1 August 2022); Sulfuric acid, 98% concentration, and Glycerol, analytical Pure, were provided by Beijing Chemical Reagent (bjhgjt.com.cn, accessed on 1 August 2022).

### 2.2. Preparation of CNC Suspension

We diluted 98% sulfuric acid to 64% and measured 350 mL of the 64% sulfuric acid into a beaker. Then, we placed the beaker on a magnetic stirrer, weighed 20 g of microcrystalline cellulose (MCC) and added it to the above. Then, we slowly added the sulfuric acid while stirring to prevent the microcrystalline cellulose from agglomerating in the sulfuric acid. The stirred suspension was poured into a round bottom flask and stirred at a constant speed in a constant temperature water bath. The microcrystalline cellulose was hydrolyzed in the acid. The reaction temperature was 40 °C, the reaction times were 2 h, 2.5 h, 3 h, 3.5 h and 4 h and the acid-concentration-to-acid ratio was 1 g:17.5 mL. After the hydrolysis reaction was completed, the hydrolyzed solution was poured into a beaker and added to a volume of 5-times ionized water, which stopped the reaction, and it was left to stand. We discarded the supernatant, centrifuged the remaining suspension at 8000 r/min for 5 min and washed and centrifuged it several times. Once we took it out, we put it into a dialysis bag with a molecular weight of 8000–14,000 for dialysis for 3–4 days until the pH of the suspension was 5–6. Then, the suspension was taken out in a translucent state and placed in a beaker for use.

### 2.3. Preparation of CNC Films

The CNC suspension prepared above was concentrated to a mass fraction of 2.3 wt%, and the cells were sonicated with 200 W power in an ice-water bath. The sonication times were 1 min, 3 min, 5 min, 7 min, 10 min, 15 min and 20 min. Then, we took 3.5 mL from the CNC suspension at different ultrasonic times and added them into a plastic Petri dish with a diameter of 3.5 cm, and the CNC film was prepared by self-assembly.

### 2.4. Preparation of CNC/PEG Composite Films

By mixing the aqueous CNC suspension with a mass fraction of 2.3 wt% that has been treated with cell ultrasonic for 1 min and PEG aqueous solution with a mass fraction of 5 wt%, the mass ratios of the CNC suspension to the PEG aqueous solution were 10:0, 9:1, 8:2, 7:3 and 6:4. After mixing, it was further stirred at room temperature for 24 h. Then, 3.5 mL of the mixed solution was poured into a plastic Petri dish with a diameter of 3.5 cm, and the CNC-PEG composite film was prepared by self-assembly.

### 2.5. Preparation of CNC/Glycerol Composite Films

Next, 0 mg, 9 mg, 15 mg, 21 mg, 30 mg and 45 mg of glycerol were added to the aqueous CNC suspension (3 g) with a mass fraction of 2.3 wt%, respectively, and mixed at room temperature for 12 h. Then, the mixed solution was poured into a plastic Petri dish with a diameter of 3.5 cm, and the CNC-glycerol composite film was prepared by self-assembly. 

### 2.6. Characterization

The morphology of the CNC samples was studied by transmission electron microscope (TEM). A 0.001 wt % CNC aqueous suspension was deposited and stained with 2% ferric chloride on a carbon-coated grid. Specimens were observed using a Hitachi HT7700 model in a high contrast mode under a 100 kV transmission electron microscope (hitachi-hightech.com, accessed on 1 August 2022). For each formulation, the width and length of approximately 500 particles were measured from the TEM image using ImageJ. A VarianCary50BIOUV visible spectrophotometer was used to record the ultraviolet–visible spectrum of the composite films in a wavelength range of 200–1100 nm (varianinc.com, accessed on 1 August 2022). The transmission spectrum was collected by installing a separate FLMS so that the surface of the FLMS was perpendicular to the beam path. We measured the FLMS in at least 10 different locations. The Hitachi S-4800 was used to observe the cross-sections of CNCs, CNC-PEG and CNC-glycerol composite film by FE-SEM. The working distance of the composite FLMS was 1 kV, and the probe current was 10 µA. The fractured sample was clamped in a thin sample, the mounting bracket was separated, and a 5 nm gold-palladium coating was applied using an agar HR sputtering coater. The composite membrane was observed on a POM microscope BX-51 (olympus.com.cn, accessed on 1 August 2022).

## 3. Results

### 3.1. Characterization of the CNC Suspension

**Figure 1:**
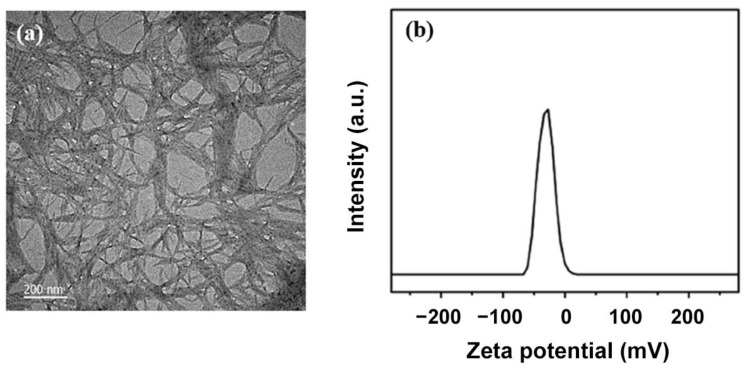
(**a**) TEM image of the as-prepared CNC; (**b**) Zeta potential of the CNC suspension.

Herein, a CNC suspension was prepared using the acid hydrolysis approach. As determined by TEM, the as-prepared CNC is rod-shaped with a length between 200 and 300 nm and a diameter within 20–30 nm, respectively (Figure 1a). The polydispersity index (PDI) of the CNC suspension is concentrated at about 0.4, with good dispersibility and uniform size. The zeta potential is one of the important indicators of the stability of the suspension. The absolute value of the potential of the CNC suspension prepared in this experiment is maintained at about 45 mV (Figure 1b), which is a significantly higher value than previously reported [2], indicating that the suspension is very stable. At the same time, the presence of negatively charged sulfate surface groups on the prepared CNC particles results in a negative potential.

**Figure 2:**
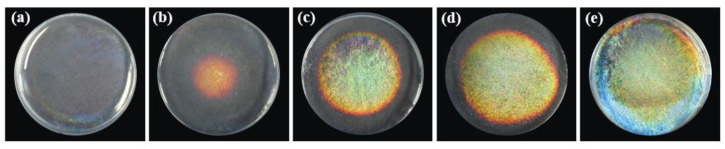
Photos of CNC film formation process: (**a**) 0 h; (**b**) 48 h; (**c**) 49 h; (**d**) 50 h; (**e**) 51 h (d = 3.5 cm).

For the self-assembly process in the CNC suspensions, when the solution concentration is low at the beginning, CNC molecules are stably dispersed in the aqueous solution with a disordered arrangement, and the solution does not show structural color at this time (Figure 2a). It continues to stand at room temperature, and with the evaporation of water, the CNC molecules begin to arrange in an orderly manner and self-assemble to form the chiral nematic phase. At first, the pitch is large. However, with further evaporation of the solvent and when it is left at room temperature for about 48 h, the pitch of the spiral structure decreases to a certain extent, the reflection wavelength is in the range of visible wavelengths and the structural color can be observed to start to appear in the solution (Figure 2b), which is yellow at this time. With the evaporation of water and further self-assembly of CNC molecules, the region forming the chiral nematic phase increases, the pitch continues to decrease, and the reflection wavelength shifts to blue. When the resting time is 51 h, the water is completely evaporated and the chiral nematic phase is also fixed in the CNC self-assembly films, at which time the pitch and the reflection wavelength are fixed.

### 3.2. Characterization of CNC Films

**Figure 3:**
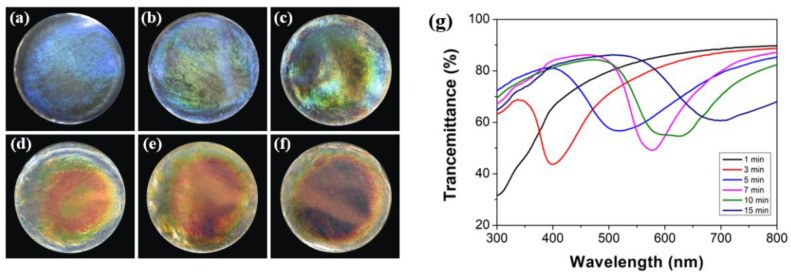
Photos of CNC films treated with different ultrasonic time (**a**) 1 min; (**b**) 3 min; (**c**) 5 min; (**d**) 7 min; (**e**) 10 min; (**f**) 15 min (d = 3.5 cm); (**g**) Transmission spectra of CNC films treated with different ultrasonic times.

The pitch of the CNC film can be controlled by subjecting the CNC suspension to different times of ultrasonic treatments, thereby controlling its reflection wavelength. As the ultrasonic time increases (Figure 3a–f), the color gradually changes from blue to red and becomes progressively more vivid. The corresponding transmission spectra show the transmittance of CNC film at a 90-degree incident angle. With the extension of the ultrasonic treatment time (1–15 min), the wavelength of the film at the maximum reflectance shifts from 300 nm to 700 nm, and the reflection bandwidth increases. Since CNC nanorods have negatively charged groups on their surface, electrostatic repulsion causes them to be uniformly dispersed in water. Some counter ions that are not completely dialyzed after acidolysis are bound in the surface layer of CNC nanorods, and the presence of these counter ions shields the surface negative charge of CNC. When no ultrasonic treatment is performed, CNC performs self-assembly with less electrostatic repulsion, which makes the pitch of the cholesteric structure relatively short. After ultrasonic treatment, energy is gradually input into the system so that some counter ions in the surface layer of CNC nanorods can leave and diffuse into the suspension. The shielding effect is weakened, and the electrostatic repulsive force of the CNC self-assembly process is larger, which enlarges the pitch of the CNC cholesteric structure. Interestingly, as the ultrasound time continues to increase, the reflected wavelength exceeds the visible range. The results show that proper sonication (200 W, <15 min) is beneficial for the preparation of uniform and bright colored films, while ultra-high sound energy input (high sound power and extended ultrasound time) has adverse effects.

### 3.3. Characterization of CNC/PEG Films

**Figure 4:**
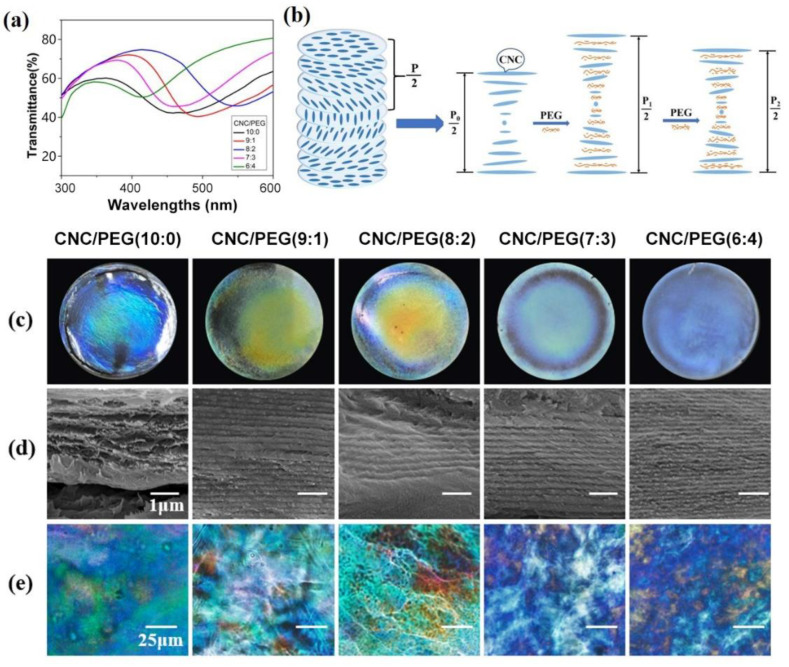
(**a**) Transmission spectra of CNC-PEG composite films; (**b**) schematic diagram of the effect of the introduction of PEG on the pitch of the CNC-PEG composite films with a chiral nematic structure. The addition of PEG causes the pitch to increase and then decrease; (**c**) photographs of the CNC-PEG composite films under natural light; (**d**) corresponding cross-sectional SEM images of the CNC-PEG composite films; (**e**) POM images of the CNC-PEG composite films.

However, the brittle nature of neat CNC film limits its applications. To improve the flexibility of CNC film, the PBG was introduced into the CNC suspension that had been properly ultrasonic treatments. In addition, the pitch of the cholesteric structure can be regulated by controlling the amount of PBG, which means the structure color of the film can be adjusted. With the increase in PEG content, the color of the composite film first turns yellow and then blue. The color change in the CNC-PEG composite film with different mass ratios was analyzed by transmission spectra (Figure 4a). The reflection wavelength of the composite film ranges from 450 nm to 550 nm, depending on the different CNC-to-PBG ratios. As the PEG content increases, the reflection wavelength first shifts to red and then shifts to blue. Interestingly, the CNC-PEG composite film shows a very uniform color, except for the edge effect of the film affected by the coffee ring [27].

To understand the relationship between the structure and color of CNC-PEG composite film with different mass ratios and to clarify the mechanism of controllable iridescence, SEM and POM are performed on the neat CNC and CNC-PEG films. As shown in Figure 4b, the cross-section SEM image of the composite films, all the samples show a clear layered structure, which represents that the introduction of PEG did not disrupt the structure of cholesteric. The distance between neighboring layers is equivalent to one-half of the pitch; therefore, we can calculate the pitch of these composite films as 220, 260, 340, 210 and 190 nm, respectively, which indicates that the change in color in composite films with different PEG content is related to the change in the pitch of the layered structure. When the mass ratio of CNC to PEG is 9:1 and 8:2, PEG can easily penetrate the nematic layer and act as a homogeneous dispersant for CNC due to the similar multihydroxy pendant groups [28]. As a result, the distance between adjacent nematic layers was enlarged, which eventually led to an increase in reflection wavelength. In this case, the CNC nanorods maintain the balance between the electron repulsion generated by the negative surface charges and the hydrogen bonding attraction caused by multihydroxyls. However, with the increase in PEG content, the penetration of excess PEG chains into the nematic layers can break the original equilibrium, thus disrupting the original long-range orientation of the mesophases of CNC. At this time, the PEG is also involved in the structure of the cholesteric phase, and a new balance is established, which will reduce the pitch (Figure 4b). As the POM images show, due to the lack of control over the spiral orientation during evaporation, there is strong birefringence and multiple color regions with random sizes and orientations in CNC films without PEG. The CNC-PEG composite films also show very strong birefringence, which also indicates the cholesteric phase structure. Moreover, the color becomes more uniform as the PEG content increases.

**Figure 5:**
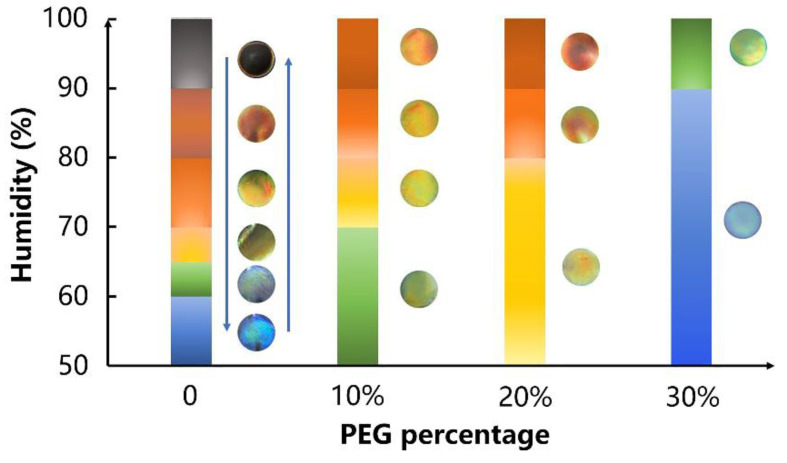
Schematic diagram of the humidity response of CNC-PEG composite films. The left side of each column corresponds to the color of the films.

The CNC and CNC-PEG composite films exhibit response performance to humidity (Figure 5). The CNC films without the PEG addition exhibit clear and rich color shifts when the RH increases from 50% to 100%; the color of pure CNC films changes from blue to cyan, green, yellow, red and, finally, transparent. At a high RH level, the color of CNC films becomes red and even transparent due to water penetration, indicating the red-shifted structural coloration. When the RH gradually decreases from 90% to 50%, the color of the film changes from transparent to red, yellow, green, cyan and, finally, returns to the original blue color, which indicates the chromism is reversible. The CNC-PEG composite films also have similar humidity response properties. As shown in Figure 5, for the composite films with 10% PEG addition, the structure color changes from green to red when the RH is 70%. When the PEG addition is 20%, the color of the composite film changes from yellow to red at 80% RH. Additionally, when the CNC-PEG composite film (7:3) was exposed to cyclic humidity, the color only changes when the RH increases to 90%, and the color changes from blue to green. From the above, with an increasing PEG ratio in the composite films, the sensitivity for the humidity-responsive performance reduces, and the color changing diminishes.

### 3.4. Characterication of CNC/Films

**Figure 6:**
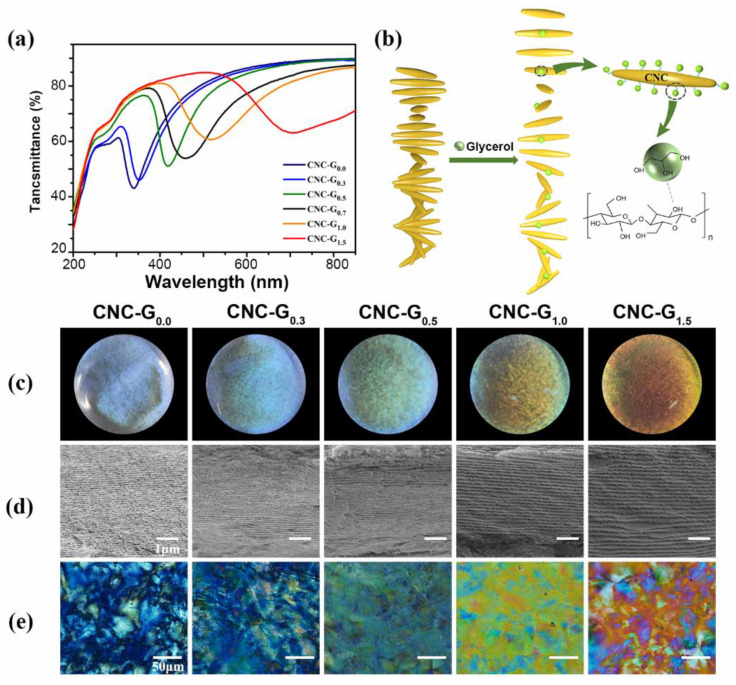
(**a**) Transmission spectra of CNC and CNC-glycerol composite films; (**b**) schematic diagram of CNC-glycerol composite films with a chiral nematic structure; (**c**) photographs of CNC-glycerol composite films. Under natural light (d = 3.5 cm), (**d**) the corresponding cross-sectional SEM images of the CNC-glycerol composite films and (**e**) POM images of the CNC-glycerol composite films.

By introducing glycerol into the aqueous CNC suspension, it is also possible to modulate the pitch and thus control the structural color. The CNC-glycerol films were prepared by introducing glycerol with different ratios into the CNC suspension. The SEM characterization and POM characterization are carried out, and the results are shown in Figure 6. As can be seen from the SEM section diagram, the composite films with different glycerol additions have a good helical layered structure. The pitches of the composite film are 260 nm, 330 nm, 360 nm, 470 nm and 600 nm. In accordance with the addition of glycerol, the pitch of the composite film increases. All the composite films have strong birefringence, which can be seen from the POM images, indicating that the addition of glycerol did not destroy the chiral nematic structure. In addition, the color of the composite films also shifted to red, as shown in Figure 6c. This is mainly because glycerol can adsorb on the surface of CNC molecules and enter the periodic layered structure of CNC film (Figure 6b), which makes its pitch increase and the reflection wavelength shift to red.

**Figure 7:**
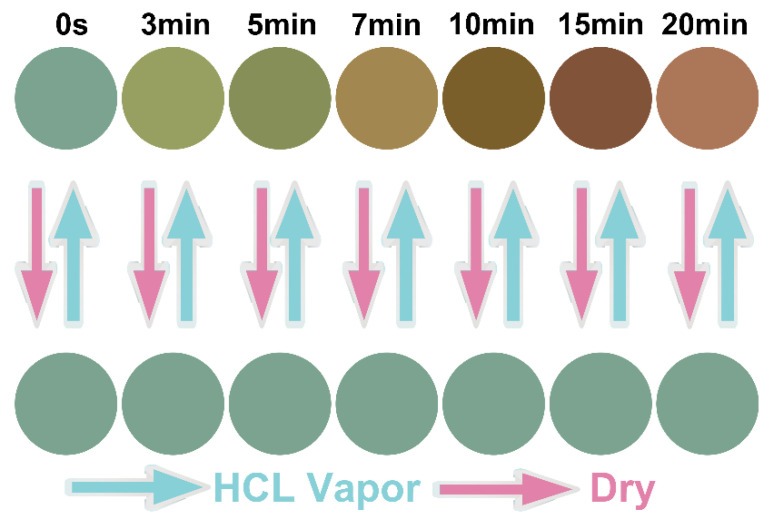
Reversible stimulation response to hydrochloric acid gas of CNC-glycerol composite films. The color change in the films under different times of hydrochloric acid gas stimulation was demonstrated. After drying, the film can return to its original color.

According to the hygroscopic property of glycerol, the stimulation response performance of CNC-glycerol composite films to hydrochloric acid gas was investigated. Taking CNC-G_0.5_ as the representative, it is stored in a closed space filled with hydrochloric acid gas for different periods of time (Figure 7). We can see that with the increase in the placement time, the structure color of the composite film CNC-G_0.5_ became red. After removing hydrochloric acid gas and drying the CNC-G_0.5_ composite film, the composite film can recover from red to its original color. CNC-G_0.5_ composite film has a reversible stimulation response to hydrochloric acid gas and can be repeated many times, so the reversibility is good. The red-shifted structural color of CNC-glycerol composite film is mainly because when the composite film is in a space filled with hydrochloric acid gas, the molecules of hydrochloric acid gas can diffuse into the periodic lamellae of CNC-glycerol composite film, resulting in the expansion of the spiral lamellae and an increase in the pitch, which makes the reflection wavelength and structural color red-shift. When the hydrochloric acid is removed, the hydrochloric acid gas molecules entering the composite film diffuse out, causing the pitch of the film to decrease and the reflection wavelength and structure color to recover.

## 4. Conclusions

In summary, stimulus-responsive and iridescent CNC-PEG and CNC-glycerol composite films were successfully prepared. Additionally, their pitch could be adjusted by subjecting the CNC suspension to different lengths of ultrasonic treatments and changing the amount of additives. The structure color covered the entire visible spectrum. By evaporating the CNC suspension, the self-assembled chiral nematic phase structure could be retained in the film, showing vivid color in a certain wavelength range due to the Bragg reflection. To this end, the conditions for preparing CNC film were explored, and a CNC suspension with a concentration of 2.3 wt% was selected and slowly evaporated at room temperature. The ultrasonic treatment and the addition of PEG or glycerol could regulate the pitch and thus control the reflection wavelength. Due to the reversible swelling and dehydration of the chiral nematic phase structure, when the RH was between 50% and 100%, the CNC-PEG composite film showed reversible structural color changing. By changing the composition of CNCs and PEG, the film could produce different humidity response properties. The CNC-glycerol film has a reversible stimulation response to hydrochloric acid gas, and similar to CNC-PEG film, it presented a stimulus response of diversified color changes by simply adjusting the additive content. This CNC-based low-cost responsive photonic material will have important applications in colorimetric biosensors, optically active ingredients, inks and decorative coatings.

## Data Availability

Not applicable.
